# Implementation of Community Participation Practices in Coordinated Specialty Care: Insights from a Qualitative Study

**DOI:** 10.1007/s10597-025-01554-w

**Published:** 2025-10-30

**Authors:** Elizabeth C Thomas, David Shern, Crystal M. Slanzi, Zahrah Surratt, Nina Bertolami, Mark S. Salzer

**Affiliations:** 1 College of Public Health, Temple University, Philadelphia, PA USA; 2 National Association of State Mental Health Program Directors, Alexandria, VA USA; 3 Department of Psychology, California State University Los Angeles, Los Angeles, CA USA; 4 College of Medicine, Drexel University, Philadelphia, PA USA

**Keywords:** Education, Employment, First-episode psychosis, Implementation science, Recovery, Young adults

## Abstract

Community participation is critical for health, particularly during young adulthood. Coordinated Specialty Care (CSC) programs for young adults with early psychosis aim to promote community participation; however, research on practices beyond those focused on employment, education, and family relationships is limited. This qualitative study explored CSC programs’ community participation practices across a broad range of areas and the factors influencing their implementation. Following a national survey, individual semi-structured interviews were conducted with 11 CSC program leaders to examine ten specific practices. Recorded responses were transcribed and qualitatively analyzed using an integrated thematic analysis approach. Findings revealed that programs implemented a range of practices, including outreach to mainstream organizations, fostering mutual support among clients, facilitating independent community participation, and providing targeted support in spirituality/religion, intimate relationships, and civic engagement activities. Effective leadership, organizational culture, and staff factors (e.g., knowledge, skills, supportive attitudes and behaviors) emerged as key facilitators, while primary barriers included limited resources, staff challenges (e.g., lack of training), and environmental challenges (e.g., stigma, rural settings). Client-level factors, such as motivation and interference from symptoms, and the perceived effectiveness and acceptability of various practices, also impacted implementation. Recommendations include enhancing leadership buy-in, staff training, and creative resource use to overcome barriers and sustain community participation efforts. This study highlights variability across CSC programs and provides actionable strategies to strengthen community participation practices for young adults with early psychosis.

## Introduction

The World Health Organization’s International Classification of Functioning, Disability, and Health (World Health Organization, 2001) defines *community participation* as a vital component of health, marked by “involvement in a life situation” across several key domains. These domains include major life areas (e.g., employment, education), community, social, and civic engagement (e.g., participation in faith communities, voting, leisure activities), and interpersonal interactions and relationships (e.g., family relationships, friendships). For individuals with serious mental illnesses, community participation is regarded as a “medical necessity” due to its strong links with positive physical, cognitive, and mental health outcomes (Salzer, 2021). This participation is especially critical during emerging and young adulthood (Arnett, 2000; Martel & Fuchs, 2017), as it influences identity development, goal-directed behavior, and the development of competence and self-efficacy (Bandura & Schunk, 1981; Garcia-Poole et al., 2018; Holland & Lachicotte, 2007). However, young adults with serious mental illnesses frequently encounter barriers to participation, including psychological issues (i.e., anxiety, low self-confidence), areas for skill development (e.g., limited social skills), and diminished access to resources (Brooke et al., 2020; Gmitroski et al., 2018; Pillay et al., 2018). Given the potential long-term impacts of experiences during this developmental stage on health and well-being (Embrett et al., 2015), fostering community participation is a critical priority for mental health programs serving young adults.

In the United States, Coordinated Specialty Care (CSC) has become the preferred approach for supporting young adults in managing early experiences of psychosis (Heinssen et al., 2014; Read & Kohrt, 2022). These early intervention programs are designed to provide developmentally appropriate services, including those that are explicitly focused on promoting community participation. For example, CSCs typically include components that support employment and education, which are widely acknowledged as key areas of interest for this demographic (Kim et al., 2019; Lucksted et al., 2015), and, among others things, might enhance their engagement in services. In addition, many CSC programs offer family psychoeducation and support services to strengthen family relationships (White et al., 2015). By addressing the needs of young adults with early psychosis in these areas, CSC programs not only enhance individual recovery outcomes but also contribute to improved community inclusion and quality of life.

Notably, the community participation interests of young adults with serious mental illness extend beyond the domains of employment, education, and family relationships. Many express a desire to engage in other aspects of community life, including the arts, spirituality/religion, sports, civic engagement, and friend and intimate relationships (Thomas et al., 2017, 2020). However, there is limited understanding of whether CSC programs promote participation across these diverse areas. Additionally, limited research exists regarding program features and practices that might enable CSC programs to effectively support community participation in a broad range of areas. Previous research (Thomas et al., 2022) conducted with young adults with early psychosis, family members, and experts in the field, identified a list of characteristics that CSCs might adopt to enhance community participation. These encompassed several key elements: specific program policies, such as a clear mission statement that explicitly prioritizes community participation across a broad range of areas; the composition, expertise, and activities of staff, including discussions about their own community participation with clients; and targeted interventions and activities, such as providing services in community-based settings and encouraging clients to support each other in their community participation. This work represented a crucial step toward understanding the ways in which CSCs may further expand and promote participation.

Understanding the factors influencing the implementation of community participation practices within CSCs is an essential next step. In a systematic review of implementation science models, Chaudoir et al. (2013) identified a five-factor framework affecting the implementation of health innovations, encompassing environmental/structural, organizational, provider, client, and innovation-level drivers. Environmental/structural-level factors refer to elements of the broader sociocultural or community context, often called the “outer setting,” in which an organization operates. These include features such as the physical environment, the political, social, or economic climate, as well as public policies and infrastructure. The organizational level includes aspects of the organization that affect implementation of a practice, including leadership effectiveness, organizational culture or climate (i.e., organizational norms and values), and resources. Provider-level factors involve characteristics of individual treatment team members, such as their attitudes, skills, and behaviors, that influence the implementation of a practice with clients. Clients’ own characteristics also moderate service engagement and intervention effectiveness, and thus the client level includes factors such as beliefs, motivation, and personality traits. Finally, the innovation level addresses the attributes of a practice itself, such as its perceived effectiveness and acceptability. This framework provides a comprehensive lens for examining diverse implementation factors.

Considering community participation practices within CSC programs, this framework can be applied to identify potential implementation facilitators and barriers. At the structural level, factors such as the availability of accessible public transportation, local employment opportunities, and stigma surrounding mental health within the broader community could impact success (Powell et al., 2021). Organizational factors might include the availability of sufficient funding to hire dedicated staff who can focus on promoting community participation, and organizational leadership that prioritizes community participation as a goal of care (Dixon, 2017; Powell et al., 2021; Stokes et al., 2022). A provider characteristic that may influence the implementation of community participation practices within CSCs is the degree to which staff believe in the importance of community participation and have been trained in interventions that promote this outcome. For example, occupational therapists possess specialized expertise in assessing and addressing personal and environmental barriers to community participation, helping individuals with mental illnesses engage in meaningful life roles (Thomas et al., 2022). At the client level, factors such as a young adult’s interest in and confidence regarding participation are important to consider (Jordan et al., 2018). Finally, an example of an innovation-level factor within CSC is the perceived value of evidence-based practices related to community participation. Supported employment and education services, for example, have been widely adopted due to evidence of effectiveness in enhancing work and school participation (Rosenheck et al., 2017) and their strong acceptability among young adults (Kim et al., 2019; Lucksted et al., 2015).

This study aimed to identify practices that CSC programs are implementing to promote community participation, partly to inspire similar approaches among other programs, and to explore the facilitators and barriers to their implementation. Given the limited knowledge in this area, qualitative methods were used to gain an in-depth understanding of the experiences of CSC teams about their efforts to promote community participation in a broad range of areas, aligning with an interpretive/constructivist epistemological approach (Merriam, 2009).

This work was part of a larger mixed-methods study examining community participation practices within CSC programs. A quantitative survey provided context for the qualitative inquiry. As part of a developmental process, an advisory group of young adults clients, family members, and professionals in the CSC field first generated a list of community participation practices within CSCs using a modified e-Delphi approach (Thomas et al, 2022) . Then, between February and April 2023, this list was used to design and administer a national survey to 30 senior leaders (i.e., CSC program directors or team members with comprehensive knowledge of their programs), recruited through a listserv affiliated with the National Association of State Mental Health Program Directors (NASMHPD) and other channels. Senior leaders were targeted given their broad oversight and ability to provide informed perspectives on program practices and factors affecting their implementation.

Building on this survey, qualitative findings captured promising practices, gaps, and modifiable barriers, providing critical insights to enhance the implementation of community participation efforts within CSCs.

## Methods

This paper primarily reports on the qualitative component of the mixed-methods study, in accordance with Standards for Reporting Qualitative Research (SRQR; (O’Brien et al., 2014). The survey described above also served as the basis for recruiting participants for follow-up interviews. As part of the informed consent process, respondents were notified that they would be contacted for an individual follow-up qualitative interview. Using criterion-i purposive sampling (Palinkas et al., 2015), survey respondents were re-contacted and invited to participate in these interviews via videoconference.

The survey collected demographic information from respondents and assessed whether CSC programs were implementing the identified community participation practices. Table 1 presents these survey findings, which provided the foundation for identifying ten practices to probe further during interviews. These practices were reportedly implemented by fewer than 70% of programs. By prioritizing practices that were less widely implemented, interviews especially sought to uncover examples of innovative approaches, potential barriers to implementation, and strategies for increasing adoption.

**Table 1:** Results from National survey of CSC programs (*N* = 30)

Community Participation Practices	*N* programs implementing practice	% programs implementing practice
Our CSC program has a mission, policy, or other statement that explicitly promotes participation in a broad range of areas as a central goal of the CSC program.	25	83
Our CSC program mission/policy or other statement is actively shared with clients, family members, and other key stakeholders.	23	77
Our CSC program routinely reviews and monitors program policies and services to ensure that they are consistent with promoting participation in a broad range of areas.	26	87
Our CSC program staff are connected to mainstream community organizations (i.e., non-mental health or social service agencies), such as community centers, faith communities, volunteer organizations, and colleges and universities. Staff are familiar with available resources, know people in these organizations, and are able to address barriers to participation, such as getting reduced memberships for program clients.	20	67
Our CSC program has an identified committee/workgroup of staff and program clients who review and discuss programmatic efforts around promoting participation in areas such as spirituality/religion, leisure/recreation, volunteering, and civic engagement.	10	33
Our CSC program actively works to address barriers to participation that our clients face, such as poverty, access to community resources, transportation, and prejudice and discrimination in the community.	28	93
Our CSC program actively promotes awareness among clients, family members, and others about the importance of community participation. Awareness efforts go beyond raising awareness about stigma.	26	87
Most staff recognize the importance of community participation to the physical, cognitive, and mental health and wellness of program clients.	30	100
Most staff have knowledge of community participation opportunities and resources that are available in clients’ communities.	29	97
Most staff understand that the environment (e.g., limited access to transportation and resources, stigma, poverty) impacts participation and attempts to address these environmental barriers.	29	97
Most staff are able to identify and address their concerns regarding community participation, such as concerns about clients’ readiness for participation or risks associated with participation, such as stress or vulnerability to being treated poorly by others.	29	97
Our CSC program educates family members, friends, and others about the importance of community participation so they can support their loved ones in increasing participation.	28	93
Most staff discuss their own community participation (e.g., their own experiences of going to church, engagement in leisure activities) with clients, including things that help them do these things and things that make it difficult to participate.	16	53
There are continuing education/professional development trainings that explicitly focus on strategies for improving community participation.	14	47
CSC program leaders communicate to staff that the community participation of program clients is important.	25	83
Our CSC program engages in active outreach to mainstream community organizations (e.g., faith leaders, parks and recreation) to identify opportunities for participation and/or reduce barriers to participation.	17	57
Our CSC program routinely assesses the participation interests of program clients beyond employment, education, and family relationships. We also ask about dating, parenting, leisure, faith interests, etc.	26	87
These assessments of community participation are used to develop service/treatment goals and steps to achieve those goals specifically related to community participation.	23	77
Our CSC program actively encourages program clients to support one another regarding community participation. This could include an ongoing group where clients discuss their participation goals, abilities, and interests and strategies for supporting one another in achieving their goals.	16	53
Some of our CSC program activities (i.e., 1:1 interactions with staff; group meetings) routinely occur in community-based settings (libraries, churches) rather than the program site or client’s home. This refers to activities other than group outings for leisure purposes (e.g., a picnic, going to a movie theater as a group).	18	60
Our CSC program offers services that address individual needs that may be directly related to enhancing a client’s community participation, such as social skills training, problem-solving training, cognitive remediation, etc.	29	97
Our CSC program promotes the development and engagement of natural supports (i.e., family members, friends, and other unpaid persons) in clients’ lives as part of promoting community participation.	30	100
All program staff (psychiatrists, social workers, peer specialists, occupational therapists, etc.) actively promote community participation in their service provision.	27	90
Our CSC program holds informational open houses/workshops to promote participation, such as “Engaging in faith communities.”	6	20
Program activities are designed to immediately promote community participation of clients on their own or with family or friends rather than starting with participation only with staff, including peer support staff.	18	60
Our CSC program routinely participates in, or organizes, events that are not just for people with mental illnesses (e.g., neighborhood clean-up).	6	20
Our CSC program routinely makes clients aware of opportunities for engaging in meaningful activities (e.g., recreation, volunteering), including low-cost or free activities	24	80
Our CSC program provides programmatic supports related to the following domains of community participation:		
Employment	30	100
Spirituality/religion	8	27
Education	30	100
Leisure/recreation	26	87
Volunteering	23	77
Relationships with parents	27	90
Sibling relationships	19	63
Friendships	28	93
Parenting (among program clients)	12	40
Dating/intimate relationships	19	63
Civic engagement	11	37

The interviewer, a trained postdoctoral fellow with expertise in qualitative methods and community participation, utilized a semi-structured qualitative interview guide to ask participants to elaborate on practices implemented by their programs and to describe environmental/structural, organizational, provider, client, and innovation-level factors that facilitated or hindered implementation. Interviews lasted between 30 and 60 min, and were audio-recorded and transcribed within the videoconference platform. Transcripts were then proofread for accuracy by the fourth author (a trained research assistant). Participants who completed interviews received a $25 electronic gift card as compensation. Interviews were conducted between June and August 2023. This study was approved by the Institutional Review Board of the first author’s institution.

### Data Analysis

To analyze the qualitative data, we employed an integrated thematic analysis approach that combined inductive and deductive coding of participants’ transcripts (Bradley et al., 2007). Two elements of the transcripts were analyzed using independent coding processes: (1) examples of how community participation practices were implemented, and (2) implementation factors impacting these practices.

The first author (an expert in CSC and community participation) conducted a detailed review of interview transcripts and identified and extracted examples of how programs implemented various community participation practices, organizing them into preliminary categories. Over the course of several meetings with the first author, the senior author (also an expert in community participation and community mental health services) reviewed these examples and categories to ensure clarity, consistency, and accuracy. Minor revisions were made to the list of examples and coding categories following this review, including the removal of examples that did not align with the conceptualization of a practice and the consolidation of overlapping categories.

The analysis of implementation factors followed a separate structured coding process. To ensure methodological rigor and enhance credibility, dependability, and confirmability (Padgett, 2012) the analysis included multiple coders and was reviewed by the senior author. The first author initially drafted preliminary coding categories, which were organized using Chaudoir et al.’s (2013) implementation science framework. Then, using this list of coding categories, the first and fourth authors independently coded each transcript. They met weekly to resolve discrepancies through discussion, which occasionally led to the development of new codes or the consolidation of existing ones. The team also engaged in critical reflection on their own assumptions by sharing these explicitly and discussing differing perspectives in coding meetings to promote reflexivity in the analysis process. Following the development of the final code list, the research team conducted a verification process to ensure the accuracy and consistency of coding across all interviews. The finalized code list, along with illustrative excerpts, was subsequently reviewed by the senior author, who confirmed the adequacy of the coding framework without recommending further revisions.

## Results

### Sample Characteristics

Of the 30 senior leaders who participated in the CSC national survey, 11 participated in a follow-up qualitative interview. Each participant represented a unique CSC program. Throughout the research process, we carefully considered “information power” and determined that the final sample size was sufficient to address the primary research questions. This conclusion was based on the study’s focused scope, rich interviewer-participant dialogue, and theory-guided analytic procedure (Malterud et al., 2015). The characteristics of qualitative interview participants, and their affiliated CSC programs, are presented in Table 2. As shown, the majority of participants were female, had over six years of experience in their current roles, and primarily served in case management roles or represented the field of social work. Participants were affiliated with CSC programs spanning seven states, reflecting diversity in both the number of clients served and the program’s duration of operation.

**Table 2:** Qualitative interview sample characteristics (*N* = 11)

	*n*	%
Gender		
Male	3	27
Female	8	73
Length of Employment		
4–6 years	4	36
Greater than 6 years	7	64
Professional Discipline		
Psychologist	2	18
Case Manager or Social Worker	5	46
Occupational Therapist	1	9
Other (e.g., LMHC)	3	27
Program Location		
Arkansas	1	9
California	1	9
Illinois	1	9
Michigan	1	9
Oklahoma	1	9
Oregon	1	9
Pennsylvania	5	46
	**M**	**SD**
Number of Clients	265	676
Age of Program (years)	14	14

### Examples of Community Participation Practices

The specific community participation practices that were the subject of qualitative interviews, along with examples from programs implementing these practices, are presented in Table 3. All programs implemented multiple practices. One program implemented two practices, two implemented four, two implemented five, two implemented seven, two implemented eight, and two implemented nine practices. No programs implemented three, six, or all ten practices.

**Table 3:** CSC programs implementing practices

Community Participation Practice	Examples	Implementation Facilitators and Exemplar Quotes	
CSC program has an identified committee/workgroup of staff and program clients who review and discuss programmatic efforts around promoting participation (*N* = 4)	1. Young adult leadership council 2. Alumni group 3. Client groups/committees 4. Behavioral health advisory committee	**Organizational/Program** Leadership effectiveness/support; culture/climate; effective communication or collaboration among staff; access supports/availability of staff or other resources; consistency of practice with fidelity model	*“I think the biggest thing is just the fact that’s built into what the model is supposed to look like from its foundation. It’s designed around getting that feedback and ownership with the clients that we work with.” (#70)*
		**Staff** Supportive attitudes/beliefs; supportive behaviors	*“It’s important to us that we get our clients involved*,* and if they are interested*,* we definitely want to really be able to enable them as much as possible to be a part of that.” (#70)*
		**Client** Motivation/interest	*“And certainly if somebody has a particular interest*,* they’re gonna participate…” (#85)*
		**Innovation** Perceived acceptability/desirability	*“We just knew was the best thing to do…For providing services to a group of people*,* we should have that group of people inform the services that we’re providing…that’s the best practice.” (#62)*
CSC program engages in active outreach to mainstream community organizations to identify opportunities for participation and/or reduce barriers to participation (*N* = 4)	1. Program provides outreach to community organizations to facilitate participation opportunities	**Environmental/Structural** Political, economic, or social climate	*“I think when people in the community hear about who we are and what we do most of the time if they can help they’re actually very generous…very open.” (#82)*
		**Organizational/Program** Leadership effectiveness/support; culture or climate; access supports/availability of staff or other resources; consistency of practice with CSC fidelity model	*“…it was a fidelity requirement for our state to do [outreach] once a month.” (#64)*
		**Staff** Skills; supportive behaviors; consistency of practice with staff training/discipline	*“There’s a lot of ways as an occupational therapist*,* she’s able to talk about these things*,* especially…to schools.” (#70)*
		**Innovation** Evidence of effectiveness	*“There’s evidence behind doing that. And so I think that supports [staff engaging in outreach].” (#91)*
CSC program staff are connected to mainstream community organizations (*N* = 10)	1. Gyms 2. Public transportation 3. GED prep courses 4. Educational institutions 5. Volunteer opportunities 6. Libraries 7. Religious organizations 9. Human services agencies 10. Employment resources 11. Community support/youth groups 12. Museums 13. Staff familiar with community resources in general	**Environmental/Structural** Political, economic, or social climate	*“In some ways the pandemic almost made it easier to connect [with community partners].” (#70)*
		**Organizational/Program** Access supports/availability of staff or other resources	*“A lot of times they would be – if somebody had burned their bridge too many times – unwilling to work with that participant. So we would have to find some kind of workaround or use funds from our agency to help those individuals.” (#64)*
		**Staff** Knowledge	*“We had such a large team at [CSC program] that…it’s a large network of people to pull from. Somebody knows somebody somewhere that we can get this thing done and achieved or access to something.” (#62)*
Some CSC program activities routinely occur in community-based settings rather than the program site or client’s home (*N* = 9)	1. CSC staff accompany clients to activities in the community 2. CSC staff provide therapeutic services in community spaces	**Organizational/Program** Leadership effectiveness/support; culture or climate; organizational or program policies; staff support; access supports/availability of staff or other resources; consistency of practice with CSC fidelity model	*“The expectation*,* I think*,* is upfront on hire that those positions are out in the community. They’re not meant to be in the clinic.” (#91)*
		**Staff** Skills; supportive behaviors	*“[A client may say] ‘I can’t meet you somewhere because I don’t want people to think I have…What if I run into someone I know?’ …Our people who are mobile do a really good job [of] putting their badge away*,* right? Maybe they’re not wearing their lanyard or the badge out. So if they’re at McDonald’s and they do see someone*,* there’s less stigma or concern attached to it.” (#73)*
Program activities are designed to immediately promote community participation of clients on their own or with family or friends rather than starting with participation only with staff, including peer support staff (*N* = 6)	1. Program emphasizes time-limited nature of supports 2. Program encourages families to participate together 3. Program helps clients build a support system 4. Program empowers clients to participate independently	**Organizational/Program** Leadership effectiveness/support; culture or climate; access supports/availability of staff or other resources; consistency of practice with CSC fidelity model	*“I think it’s just kind of built in.” (#73)*
		**Staff** Skills; supportive behaviors	*“It can be really helpful to have a hands-on support from staff when [clients] may be short with motivation or energy*,* and…that accountability can really help to where it’s something that’s scheduled rather than left up to them*,* and then it may or may not happen.” (#86)*
		**Innovation** Perceived acceptability/desirability	*“So having that mentality…espoused by the agency and then the providers within our program*,* all are pretty passionate about that. Those are really powerful tools that we have to be able to make that happen as much as we can.” (#70)*
CSC program actively encourages program clients to support one another regarding community participation (*N* = 9)	1. Group activities (in the community) offer opportunities for clients to support one another 2. CSC clients serve as mentors 3. Program offers groups around mutual interests/specific participation activities	**Organizational/Program** Leadership effectiveness/support; organizational or program policies; access supports/availability of staff or other resources	*“I think having the finances to be able to go out and do fun community activities*,* which I think generates excitement and an expectation that our clients are out in the world.” (#91)*
		**Staff** Skills; supportive behaviors	*“We have a really great staff of people who…have comfort working with groups in the community*,* which I think is also another type of skill.” (#82)*
		**Client** Motivation/interest	*“We have the participation there and the buy-in from the participants.” (#64)*
Most staff discuss their own community participation with clients… (*N* = 8)	1. Staff discuss challenges to participation 2. Peer specialists share personal experiences 3. Staff share about participation in community events/activities 4. Participation experiences come up in groups 5. Staff share participation success stories	**Organizational/Program** Leadership effectiveness/support; culture or climate; staff support	*“We know that recovery happens in relationship and that the way to build genuine relationship is to be able to share your own experiences and share of yourself in a safe way…The culture of [CSC program] created a space where that was kind of normal. And there were times where new therapists would come in and they’d be like*,* ‘I can’t share this. I can’t say that. I don’t want to overshare.’ and so it’s coaching them…around there are things that you can share and how you share things that don’t give somebody too much of insight into your personal situation*,* but can still validate their experience.” (#62)*
		**Staff** Knowledge; skills; supportive attitudes/beliefs; supportive behaviors; consistency of practice with staff training/discipline	*“We really harp a lot on that relationship building. Most of my team has social work backgrounds. So…it’s kind of instilled in us in that way.” (#73)*
CSC program provides programmatic supports related to spirituality/religion (*N* = 4)	1. Program assesses interest in spirituality/religion at intake to make it part of a focus of treatment 2. Program addresses interest in spirituality/religion as it comes up in treatment 3. Program focuses on spirituality/religion as a way to provide culturally competent care	**Environmental/Structural** Physical environment or infrastructure surrounding program	*“Because we’re…mainly rural*,* we just know so much more about the communities…it’s like ‘oh you tell me that you were raised Methodist*,* you know “Joe” down there he’s been the minister down there for a long time and you [can] check him out.’ You know*,* it’s much easier to do that than if you lived in [big city]*,* or someplace.” (#88)*
		**Organizational/Program** Leadership effectiveness/support; culture or climate; effective communication or collaboration among staff; access supports/availability of staff or other resources; consistency of practice with CSC fidelity model	*“…the fidelity model as a whole is looking at more than just getting symptoms under control*,* it’s getting people on their chosen life path. And so really encouraging a holistic approach of care and finding their place in the world around them*,* and then having an agency that’s really supportive in allowing us to make sure that we’re able to address our clients’ needs.” (#70)*
		**Staff** Knowledge	*“We’ve been kind of fortunate too because we have a two Hispanic workers on our team and so they’ve educated us as well about religion and spirituality with individuals who are Hispanic.” (#85)*
CSC program provides programmatic supports related to dating/intimate relationships (*N* = 9)	1. Assesses interest in dating/intimate relationships at intake to make it part of a focus of treatment 2. Provide specific supports related to dating/intimate relationships 3. Focus on dating/intimate relationships in groups 4. Address concerns around dating/intimate relationships as it comes up in treatment	**Organizational/Program** Leadership effectiveness/support; organizational or program policies; consistency of practice with CSC fidelity model	*“What supports us in doing this*,* part of fidelity model is assessing interpersonal relationships and where people’s needs are on that*,* what they want to do.” (#70)*
		**Staff** Knowledge; supportive behaviors	*“I think just having the knowledge of what agencies are out there…maternal health is one that has provided that educational series to us in the past. And we’re lucky enough to have an intern this year who works for an agency that*,* provides that kind of psychoeducation this year.” (#85)*
		**Client** Motivation/interest	*“Folks are willing to participate.” (#64)*
CSC program provides programmatic supports related to civic engagement (*N* = 5)	1. Program assesses interest in civic engagement at intake to make it part of a focus of treatment 2. Program supports clients to register to vote 3. Program supports clients in getting involved in volunteer opportunities 4. Program supports advocacy	**Organizational/Program** Leadership effectiveness/support; access supports/availability of staff or other resources	*“The agency is supportive.” (#70)*
		**Staff** Knowledge; supportive attitudes/beliefs	*“Just the level of interest of staff to participate in it.” (#85)*

The first practice, related to CSCs having an identified committee or workgroup of staff and program clients to review and discuss programmatic efforts around promoting community participation, was implemented by four programs. Three of these programs discussed how community participation programming was an ongoing part of discussions within existing client or former client groups, such as a young adult leadership council or alumni group. One program described having a “behavioral health advisory committee,” that included both clients and staff, although it was unclear the degree to which this committee focused explicitly on community participation beyond the behavioral health system. Two additional programs provided examples of how discussions about community participation were incorporated within routine staff interactions, such as during treatment team meetings. However, because this approach did not fully align with the advisory group’s original conceptualization of the community participation practice, it was not included as an example of its implementation.

Engaging in active outreach to mainstream community organizations was a practice implemented by four CSC programs. These programs provided clear examples of how outreach was used to identify opportunities for participation and reduce barriers to participation. For example, one program director discussed how her program connected with a local “restauranteur” to pilot a series of cooking classes for clients. Five additional programs shared examples of outreach that did not have a clear connection to promoting community participation but rather was focused on educating the community about early psychosis symptoms and treatment (e.g., *“Usually once monthly*,* we would pick an organization and I would go out and provide education…on our CSC model…I would use it as…a referral source and as a resource for us to use for our participants”* (#64)). Therefore, these examples were not considered in the analysis.

The third practice, implemented by 10 programs, related to CSC program staff being connected to mainstream community organizations accessible to anyone regardless of mental health status. A wide variety of these organizations were mentioned, including gyms (e.g., “YMCAs”), public transportation programs, educational programs and institutions (e.g., “GED prep courses,” local high schools and colleges), volunteer organizations (e.g., “SPCA”), libraries, religious organizations (e.g., “church groups”), human service agencies (e.g., for “housing assistance” programs), employment resources (e.g., “job fairs” hosted by community organizations), community support/youth groups (e.g., “LGBTQ youth organization”), and museums. Two of the 10 programs also discussed how their staff were connected to different programs (e.g., *“Our care coordinator in particular is really good about going out and just finding everything that’s out there to be able to get our clients connected to them.”* (#70)).

Nine programs endorsed implementing at least some program activities routinely in community-based settings. For example, some staff accompanied clients to activities in the community:

*“It’ll also be going out*,* and maybe they’re doing employment searches…we’ll just go out and meet downtown*,* and we’ll just walk from place to place*,* and we’ll be outside where nobody can see us. But we’ll be supporting them as they go in and ask for applications*,* or if somebody’s hiring. We’ll go to the Y.M.C.A. or a fitness club if that’s what they’re wanting.”* (#70).

Three of the nine programs provided examples of how staff meet with clients in community spaces, such as coffee shops or restaurants, for the purpose of providing CSC services (e.g., “*It’s not uncommon for us to do a session in the park with them or to take them to the library. And those are the individual sessions.”* (#85)).

Six programs reported that program activities are designed to immediately promote community participation of clients on their own or with family or friends rather than with staff. One program indicated this was accomplished by emphasizing the time-limited nature of support:

*“We really encourage everybody that we are not a permanent program. We are a up to 2 years*,* and at some point you’ll be graduated and you’ll get sick of us*,* which is great…But what we wanna make sure is going on is that you have those people that are gonna be around forever like your parents or your wife or husband…and we want them to be involved with us from the very get go…we’re really gonna encourage [family members] to be [supporting clients] instead of us*,* even if we’re happy to help out…So really making sure that they realize that we’re trying to build that independence and that creation of a support network around them.”* (#70).

Three of the six programs discussed how they encourage family members to participate in community-based activities together as a means to restore relationships (e.g., *“I think our family therapist was pretty good at doing that. Encouraging outings or events to kind of rebuild a damaged family relationship or communication challenges.”* (#73)). Another program described a focus on helping clients “expand [their] social circles” and develop deeper relationships with acquaintances (#82). Finally, program #85 emphasized the value of empowering clients to participate independently, recounting how the team peer specialist supported a client in independently navigating the public transportation system.

The practice of actively encouraging program clients to support one another regarding community participation was implemented by nine programs. Examples of this practice included organizing community group activities (e.g., *“go[ing] to the beach*,* Frisbee golfing”* (#70)) to enable mutual support and creating mentorship opportunities between clients (*“We did have somebody that took on the role of mentor…he would offer to give people rides home…they would meet…to go play video games or go play golf…”* (#70). Five of the nine programs described holding ongoing groups based on shared interests in community participation:

*“We have a rotation of groups that occur at [program] all the time…the groups are driven by participants on what their interests are…engaging around anime*,* engaging around photography*,* they did mural tours so they walked the city in different areas of the neighborhoods and talked about the murals and what they meant to them. We have chess club and writing…group and art group. There’s cooking group…whatever a young person’s passion is there’s probably space at [program] to create or to engage with other people who are interested in the same thing.”* (#62).

Self-disclosure of community participation experiences by staff with clients was discussed by eight programs. Seven of these programs talked about how staff often share their personal experiences with community events and activities, including success stories and challenges related to community participation. This practice was illustrated in a comment by one program director, *“There are times when…appropriate use of self we would do. Like*,* ‘hey I had this success*,*’ or something that’s relatable*,* and ‘in my church group we do this.’ Because my thoughts are we share the community with the folks that we serve. So why not appropriately share what has worked for us?”* (#64). Two of the eight programs indicated that these discussions tended to come up in the context of group sessions. For three programs, self-disclosure related to community participation tended to be seen as the primary responsibility of peer specialists on the team (e.g., *“I think that it comes up the most with our peer support specialist…inherently*,* that’s his role to share his experiences.”* (#73)).

Related to practices 8–10, a subset of programs reported implementing programmatic supports across three specific domains of community participation (i.e., spirituality/religion, dating/intimate relationships, and civic engagement). Four programs provided supports focused on spirituality and religion, nine addressed dating and intimate relationships, and five offered supports related to civic engagement. In some cases, this was accomplished by assessing interests and needs in these areas at intake to inform treatment planning. Programs also responded to clients who expressed these needs during the course of treatment. For example, one program director described their approach to supporting spirituality/religion:

*“So that’s something that they’re expressing interest in pursuing is strengthening their spirituality or finding a church or something then we’re happy to explore with them. We can tell them either*,* ‘Hey*,* I know that there’s these ones or…we can look up how to find them or go through with how to access those different things*,* and maybe find reviews or have a conversation of…do you know what your spirituality is - you have a chosen religion?”* (#70).

One program included a focus on spirituality and religion as a way to practice cultural competence (e.g., “*We have a lot of individuals that are Hispanic and there’s a lot of religion involved there. So we’re very mindful and…the staff have done a lot of reading in certain areas. So we’re very sensitive to what their religious beliefs are.”* (#85). Six programs incorporated dating and intimate relationships into ongoing groups. One program described offering a healthy relationships series: *“We’ve had [groups] that were 6 and 8 weeks long that…[that] talked about intimate partner violence and other ways to identify…is this a safe and healthy relationship for me? Is this not? If it’s not*,* what can we do? How can I get help? How can I get out of it?”* (#62)). Related to civic engagement, four programs provided assistance with activities like registering to vote and getting involved in volunteer activities and advocacy efforts.

### Implementation Factors

As shown in Table 3, programs that implemented the various community participation practices consistently reported effective leadership and organizational support as key implementation facilitators. Leadership effectiveness – encompassing participants’ own program leadership as well as leadership at the organizational and state levels – was pivotal in promoting practices such as having a committee to review community participation efforts, conducting outreach to mainstream community organizations, conducting activities in community-based settings, encouraging client autonomy and mutual support in community participation, fostering staff self-disclosure around community participation, and offering programmatic supports around spirituality/religion, dating/intimate relationships, and civic engagement. Organizational culture and access to resources also played a significant role in supporting many of these same practices. Staff factors, including supportive behaviors and attitudes and specific knowledge and skills, further facilitated these practices. For example, staff knowledge about community-based resources and training in particular disciplines (e.g., occupational therapy) contributed to successful outreach efforts and connection to mainstream community organizations. Environmental/structural, client, and innovation-level implementation factors were also mentioned when discussing a few of the community participation practices. The political, economic, or social climate was recognized as important for the implementation of outreach and connection to mainstream community organizations, while the physical environment was relevant for offering programmatic supports around spirituality and religion. Client motivation and interest were essential for practices like having a community participation committee and implementing mutual support around community participation. Finally, perceptions about effectiveness or acceptability were noted as relevant to the implementation of having a community participation committee, engaging in outreach, and encouraging independent participation.

In contrast, as presented in Table 4, programs that did not implement these practices frequently cited barriers such as insufficient staffing and limited resources. Environmental and structural barriers, including the political, economic, or social climate (e.g., stigma) and the physical environment surrounding the program (e.g., rural setting), limited outreach efforts, connection to mainstream community organizations, and staff self-disclosure practices. Staff challenges, including non-supportive behaviors, inadequate training, and discomfort, hindered implementation of practices like engaging in outreach, using self-disclosure, and offering programmatic supports around specific domains of participation. In particular, supporting civic engagement and spirituality/religion was often deprioritized due to perceived low client interest or staff concerns about appropriateness for clients with psychosis. Client issues (e.g., low motivation, interference from symptoms, lack of personal resources) were also noted in relation to serving on community participation committees and engaging in independent community participation. Finally, innovation-level implementation barriers (i.e., lack of perceived acceptability) affected program activities occurring in community-based settings.

**Table 4:** CSC programs not implementing practices

Community Participation Practice	Implementation Barriers and Exemplar Quotes	
CSC program has an identified committee/workgroup of staff and program clients who review and discuss programmatic efforts around promoting participation (*N* = 7)	**Organizational/Program** Lack of access/issues with staffing or other resources; issues related to telehealth	*“I think the biggest challenge is that all of the team members…we’re all part time within the CSC program because we all carry…a clinical case load*,* but only 10 hours of their week are dedicated to the CSC.” (#73)*
	**Client** Issues with motivation/interest	*“We’ve always traditionally had a really hard time engaging participants in…extracurricular stuff. Kind of the buy-in to some of those pieces has been traditionally pretty difficult.” (#73)*
CSC program engages in active outreach to mainstream community organizations to identify opportunities for participation and/or reduce barriers to participation (*N* = 7)	**Environmental/Structural** Political, economic, or social climate	*“I’ve reached out to several of the local universities and high schools*,* and I haven’t gotten really any response.” (#86)*
	**Organizational/Program** Lack of access/issues with staffing or other resources	*“A lot of it is just having time to devote to it…staff bandwidth to devote to it.” (#59)*
	**Staff** Non-supportive behaviors	*“So typically I’ll reach out and try to identify the right person to reach out to and then I’ll follow up with them. But if I don’t get a follow-up after that second time I typically don’t reach back out to them.” (#86)*
CSC program staff are connected to mainstream community organizations (*N* = 1)	**Environmental/Structural** Political, economic, or social climate	*“The topic of psychosis can be very challenging*,* whereas they - colleges*,* universities*,* high schools - maybe see depression*,* anxiety more commonly. I don’t think psychosis is in the forefront of a lot of people’s minds. It hasn’t become very normalized in our society yet. It’s very stigmatized…There’s just a lack of information*,* a lack of awareness of how much psychosis impacts individuals in our community.” (#86)*
	**Organizational/Program** Lack of access/issues with staffing or other resources	*“One of the barriers is time…there’s a lot going on in the office with the [CSC program] and making time for outreach is obviously very important but it can be challenging to develop those relationships outside of the agency.” (#86)*
Some CSC program activities routinely occur in community-based settings rather than the program site or client’s home (*N* = 2)	**Organizational/Program** Culture or climate; organizational or program policies	*“We’ve got to keep a clinic-based schedule back-to-back to keep the doors open.” (#88)*
	**Innovation** Lack of perceived acceptability/desirability	*“A lot of times we find that client engagement is greater if we go to their home rather than have them come to the community.” (#86)*
Program activities are designed to immediately promote community participation of clients on their own or with family or friends rather than starting with participation only with staff, including peer support staff (*N* = 5)	**Client** Unhelpful attitudes/beliefs; unhelpful behaviors; interference from symptoms; lack of social supports	*“I think generally speaking our participants when they first come to us are pretty acutely ill and symptomatic and not necessarily able to do that immediately. But we do have that as a long-term goal…doing more and more to them transition to on their own doing things.” (#79)*
CSC program actively encourages program clients to support one another regarding community participation (*N* = 2)	**Environmental/Structural** Issues related to weather/timing	*“A lot of times it’s because youth who are enrolled in school have a lot of commitments…right now*,* we have trouble getting youth in because you got some kids in sports*,* they’re already in summer practices 2 times a day. And so we get to work around school activities and in the other activities. So it’s been harder to pull the youth together.” (#88)*
Most staff discuss their own community participation with clients… (*N* = 3)	**Environmental/Structural** Physical environment or infrastructure surrounding program	*“What gets in the way is that we live in rural areas. So we’re always careful as clinicians not to talk very much about our own experiences in the community because it’s like*,* oh yeah*,* she’s my cousin or….And so we section it off pretty good*,* especially in the small communities.” (#88)*
	**Organizational/Program** Lack of staff support	*“We don’t set an expectation and so it ends up being what people do naturally. I do see that as something we could potentially do differently with more training or education around how to do that*,* in a way that people feel comfortable with*,* but I think we haven’t sort of prioritized making people feel comfortable enough to do that routinely.” (#79)*
	**Staff** Non-supportive attitudes/beliefs; inconsistency of practice with staff training/discipline	*“And maybe staff don’t feel comfortable. Certain staff have different levels of comfort when it comes to self-disclosure. And so that might be part of it too*,* is all staff have different boundaries when it comes to…what they feel comfortable disclosing and sharing with the client*,* how open they are…” (#86)*
CSC program provides programmatic supports related to spirituality/religion (*N* = 7)	**Organizational/Program** Culture or climate; lack of access/issues with staffing or other resources	*“I think we as a maybe clinic don’t go out of our way to really dig into clients’ religious preferences and encourage it. Some of our clients because of their psychosis actually have religious delusions. And so also there’s a really fine balance between wanting to encourage religious participation*,* but not knowing if it’s a symptom necessarily so I think…you have to be a little bit careful when it comes to religion.” (#91)*
	**Staff** Lack of knowledge	*“I just don’t think we have anybody knowledgeable enough to do that.” (#64)*
	**Innovation** Lack of perceived acceptability/desirability	*“For me and my personal experience with clients with psychosis*,* I’ve never had that question come up.” (#86)*
CSC program provides programmatic supports related to dating/intimate relationships (*N* = 2)	**Staff** Non-supportive behaviors	*“I talked about those things with clients. I think my peer talks about those things*,* when clients want to date. But I think it’s more like talking and less action. And maybe I’m probably the only one.” (#91)*
CSC program provides programmatic supports related to civic engagement (*N* = 6)	**Organizational/Program** Culture or climate; lack of access/issues with staffing or other resources	*“Anything political feels really dicey the last few years. Like we barely can handle talking about it or kind of managing it as a staff…and the thing with psychosis is that it affects everybody*,* right? So we have families of all kinds of political beliefs. And I think it’s…too dicey.” (#82)*
	**Client** Issues with motivation/interest; lack of personal resources	*“It’s like…please send someone here to the state planning group in [City]. If I’ve got a client…that’s coming to the mental health center and probably has Medicaid*,* that client is not going to be able to afford to travel…or have sufficient transportation to those activities that help them organize on the state level or even maybe at a local level. I think the resources for our clients to be involved in more political organizing or community organized advocacy…that’s been hard because they lack the resources.” (#88)*
	**Innovation** Lack of perceived acceptability/desirability	*“They’re still with day-to-day life. They’re not thinking about civic engagement*,* They’re thinking about other things like going to school*,* or education or getting these symptoms under control. Or playing video games*,* whatever it may be. I don’t know to what extent civic engagement is at the forefront of their mind to where we would focus on it.” (#86)*

## Discussion

The present study was the first to explore and describe the implementation of practices within CSC that promote community participation across a broad range of areas. The findings reveal significant variability in how these practices are applied across programs and elucidate the range of factors facilitating or impeding their implementation. Ultimately, the results offer practical examples of how CSC can expand their efforts to promote community participation and recommendations for overcoming challenges and strengthening a focus on community participation within CSC.

While the practices explored in this study were not universally implemented, they provide valuable examples of approaches that additional programs could consider adopting. These practices included establishing groups comprised of clients and/or staff to advise on participation initiatives, fostering mutual support among clients through group activities, and connecting with community resources like gyms and libraries. Staff provided real-time support by accompanying clients into the community and used healthy self-disclosure to encourage participation. Some programs also offered targeted support in areas such as spirituality/religion, dating/intimate relationships, and civic engagement. Findings underscore the importance of tailoring strategies to the unique needs and contexts of individual programs, as demonstrated by the diverse ways in which the ten community participation practices were operationalized across sites.

It is also likely that there are additional approaches that programs could implement to further enhance community participation. For example, while one program described having a “behavioral health advisory group,” it was unclear to what extent the group focused on community participation beyond the behavioral health system. Programs might consider establishing committees specifically dedicated to identifying and addressing barriers to community participation, ensuring a broader focus beyond clinical recovery. Similarly, many programs described outreach efforts in relation to educating the community about first-episode psychosis and early intervention services, which is a typical practice within CSC programs across varying models (Jumper et al., 2024). Programs could use outreach as an opportunity not only to connect clients with treatment but also to encourage their broader participation beyond the mental health system. For example, they might provide education to community organizations about the importance of community participation for everyone, including clients with early psychosis, and collaborate with them to introduce clients to participation opportunities, such as volunteering, recreational activities, job training programs, or college support services. Finally, several programs mentioned conducting therapeutic sessions in community spaces. These activities could be expanded to further support clients’ independent participation in these settings. For example, sessions held in a café or library could include guided practice in initiating social interactions or navigating public spaces, helping clients build confidence in making use of these locations independently. Similarly, conducting sessions in a gym or recreational center could involve practicing strategies for overcoming anxiety related to group activities or establishing exercise routines that clients can sustain on their own.

Implementation factors identified in this study suggest pathways for programs to enhance their focus on community participation. Strong leadership and organizational support emerged as pivotal facilitators across many of the practices, particularly in fostering a culture that prioritizes community participation and providing the necessary resources to implement practices effectively. The skills and abilities of individual staff also played a crucial role. These findings align with existing research on factors influencing the implementation of CSC services that emphasizes the importance of workforce development and organizational climate (Dixon, 2017; Powell et al., 2021; Stokes et al., 2022). There are several ways that programs could strengthen these facilitators. For example, programs could improve organizational leadership’s recognition of the value of community participation through systematically collecting and presenting client success stories and data that demonstrate its positive impact on recovery outcomes. Additionally, enhancing staff training – particularly in areas such as appropriate self-disclosure – could help increase confidence and effectiveness in implementing community participation practices. Given their relevance to specific community participation practices (e.g., outreach, community participation committees, promoting independent participation), factors such as the broader community’s receptiveness to including clients with early psychosis, client motivation, and perceptions about the benefits community participation practices can also serve as key targets to enhance implementation. For example, CSC providers might educate community organizations about the process and advantages of creating welcoming environments (Snethen et al., 2021), enabling these organizations to experience the unique talents and meaningful contributions of clients with early psychosis.

CSC programs also need to attend to the implementation barriers identified in this study. Programs that did not implement various community participation practices frequently cited issues with staffing and funding, which is consistent with challenges faced in the broader implementation of CSC services and other community mental health programs (Dixon, 2017; Hoge et al., 2013; Powell et al., 2021; Stokes et al., 2022). However, this study’s findings also highlight that these practices can be feasibly implemented, even within the constraints of limited resources and diverse organizational contexts. In addition to leveraging the facilitators of implementation identified by some programs, creative solutions - such as designating ‘community inclusion specialists’ on treatment teams, advocating for the medical necessity of community participation programming with funders, and utilizing existing community-based (vs. clinic-based) resources - may help mitigate these barriers (Baron, 2018). Environmental challenges, particularly in rural settings, may be addressed through strategies like organizing groups to assist individuals in identifying and planning participation opportunities and incorporating people with lived experience into anti-discrimination efforts (Slanzi et al., 2024). Furthermore, staff training should focus on overcoming hesitations regarding client engagement in certain areas, such as spirituality or religion, as these concerns have been identified as barriers to providing effective support (Neathery et al., 2020). Finally, client-level challenges can be addressed by adopting empowerment-focused approaches that help young people pursue personally meaningful goals and access necessary supports when needed (Klodnick et al., 2023).

### Limitations

Several limitations should be considered when interpreting this study’s findings. First, the small sample size restricts the generalizability of the results to all CSC programs. Furthermore, the programs included in this study may not fully represent the diversity of all CSCs, as those that participated may have had a particular interest or commitment to promoting community participation, which could bias the findings. However, the primary aim of this study was not to assess the overall state of community participation practices across all CSCs but rather to provide illustrative examples of these practices and the associated implementation factors. Second, due to both practical and substantive constraints, we were unable to follow up with all programs about every practice queried in the national survey, which limits the depth of understanding regarding the full range of practices and their implementation contexts. Finally, we were also unable to follow up with all programs that completed the national survey regarding their community participation practices. This incomplete follow-up means that the findings may not fully capture the experiences of all participating programs, potentially leaving out important perspectives.

## Conclusions

This study clarified how CSC programs support community participation, offering diverse examples of practices that can inspire broader adoption and innovation. The findings demonstrate the feasibility of implementing practices across a range of domains, while also highlighting variability and the importance of tailoring strategies to program-specific contexts. Strong leadership, organizational culture, and staff characteristics emerged as particularly critical facilitators, underscoring the value of investing in these areas to sustain and enhance community participation efforts. By leveraging these insights and building on identified facilitators, CSC programs have the opportunity to expand their impact, empowering young adults with early psychosis to engage more fully in their communities. Future research can further clarify the links between specific practices and participation outcomes, guiding the development of evidence-based approaches.

## Data Availability

These data will be deposited in the Interuniversity Consortium for Political and Social Research (ICPSR) repository no later than 24 months after the grant award end date, in accordance with ACL’s Public Access Plan.
